# The effectiveness of iron supplementation for postpartum depression: A protocol for systematic review and meta-analysis

**DOI:** 10.1097/MD.0000000000023603

**Published:** 2020-12-11

**Authors:** Yanran Tian, Zeyu Zheng, Chen Ma

**Affiliations:** a Obstetrics Department; b Information Section, the People's Hospital of Nanchuan, Chongqing, China.

**Keywords:** iron supplementation, meta-analysis, postpartum depression, protocol, systematic review

## Abstract

**Background:**

Postpartum depression (PPD) is one of the most common postpartum psychiatric disorders. The prevalence of PPD ranges from approximately 10% to 30%. In recent years, iron supplementation has emerged as potential means to treat PPD, and an increasing number of studies have been published to support the effectiveness of iron supplementation for PPD. we will conduct a comprehensive systematic review and meta-analysis to evaluate the evidence of randomized controlled trials for iron supplementation treatment of PPD.

**Methods:**

PubMed, Embase, Cochrane Library, Web of Science, China National Knowledge Infrastructure, China Science, and Technology Journal Database, and Chinese Biomedical Literature Database will be searched from their inception of databases to December 31, 2020. Two reviewers will select articles, extract data and assess the risk of bias independently. Any disagreement will be resolved by discussion with the third reviewer. Review Manager 5.3 software will be used for data synthesis. The Cochrane risk of bias assessment tool will be used to assess the risk of bias.

**Results:**

This study will conduct a comprehensive literature search and provide a systematic synthesis of current published data to explore the effectiveness of iron supplementation for PPD.

**Conclusions:**

This systematic review and meta-analysis will provide clinical evidence for the effectiveness of iron supplementation for PPD, inform our understanding of the value of iron supplementation in improving PPD symptoms, and help clinicians to make better decisions regarding the appropriate role of iron supplementation as a part of prevention and treatment routines.

**Study registration number:**

INPLASY2020110007

## 1 Introduction

Postpartum depression (PPD) is one of the most common postpartum psychiatric disorders.^[1]^ It is a combination of depressed mood, loss of interest, anhedonia, impaired concentration, feelings of guilt, or worthlessness and suicidal thoughts.^[2]^ Some patients often have thoughts of harming their child.^[3]^ The prevalence of PPD ranges from approximately 10% to 30%.^[4,5]^ The best predictors for PPD are the psychiatric history and adverse life events.^[6]^ Poor social support, persistent infant health problems, marital difficulties and violence are the common psychosocial risk factors.^[7–10]^ Besides, rapid changes in estrogen and progesterone during pregnancy, genetics, and immune function have been implicated in the pathophysiology of PPD.^[11–13]^ Psychological interventions such as cognitive behavioral therapy, interpersonal therapy and dynamic therapy have been specifically adapted for mild to moderate PPD.^[14–16]^ When PPD is severe or not sufficiently responsive to psychological treatment, pharmacological interventions may be necessary. The selective serotonin reuptake inhibitors as the first-line antidepressant medications for PPD have been showed higher remission rates compared to placebo.^[17]^ Electroconvulsive therapy is considered to treat severe PPD, especially in the setting of intractable suicidality.^[18]^

In recent years, iron supplementation has emerged as potential means to treat PPD because many studies have documented the association of iron deficiency anemia and low ferritin with PPD.^[19,20]^ Iron deficiency can change concentrations of cytochrome C, dopamine, serotonin, and gamma-aminobutyric, and have numerous adverse effects on brain function.^[21]^ One study showed that as iron stores decreased, risk of developing PPD increased, and many PPD patients had depleted iron stores.^[22]^ Sheikh et al found a positive association between early iron supplementation and decrease in Edinburgh Postnatal Depression Scale score, and continued PPD might be related to the lower postpartum ferritin levels in untreated mothers.^[23]^

Up to now, no systematic review or meta-analysis has been performed on the effectiveness of iron supplementation for PPD. Therefore, we will conduct a comprehensive systematic review and meta-analysis to evaluate the evidence of randomized controlled trials (RCTs) for iron supplementation treatment of PPD.

## 2 Methods

### 2.1 Study registration

This study has been registered on INPLASY (INPLASY202011000**7**). It will be performed following Preferred Reporting Items for Systematic Reviews and Meta-Analyses statement.^[24]^

### 2.2 Eligibility criteria for study selection

#### 2.2.1 Types of studies

All RCTs of iron supplementation for PPD will be included without language limitation. Case reports, animal experiments, and reviews will be excluded.

#### 2.2.2 Types of participants

Participants who meet the diagnostic criteria of PPD will be included without restrictions of nationality, age, gender, and race.

#### 2.2.3 Types of interventions

In the treatment group, patients were given iron supplementation with no limitations of administration routes, dosage, or duration of intervention.

RCTs that have control groups with conventional treatments (such as psychological interventions and pharmacological interventions) or no treatment will be included.

#### 2.2.4 Types of outcomes

Edinburgh Postnatal Depression Scale score will be designated as the primary outcome. Improvement rate will be designated as the secondary outcome.

### 2.3 Search strategy

PubMed, Embase, Cochrane Library, Web of Science, China National Knowledge Infrastructure, China Science, and Technology Journal Database and Chinese Biomedical Literature Database will be searched from their inception of databases to December 31, 2020. The detailed search strategy for PubMed is shown in Table 1. The similar search strategies will be used for other electronic databases.

**Table 1:** Search strategy for PubMed.

Number	Search terms
1	postpartum depression
2	PPD
3	postnatal depression
4	post-partum
5	post-natal
6	depression
7	Or 1–6
8	iron
9	intravenous iron infusion
10	oral iron
11	iron supplementation
12	Or 8–11
13	randomized controlled trial
14	clinical trial
15	trial
16	random
17	RCT
18	placebo
19	Or 13–18
20	7 and 12 and 19

PPD = postpartum depression, RCT = randomized controlled trial.

### 2.4 Selection of studies

The searched literature will be imported into Endnote X9 (Thomson Reuters). The duplicates will be excluded by Endnote X9. Two reviewers will independently scan titles and abstracts to eliminate all irrelevant records. After reading the full text of the remaining records, the final included studies will be determined. Any disagreement about the selection of studies will be resolved by discussion with the third reviewer. A Preferred Reporting Items for Systematic Reviews and Meta-Analyses flow diagram will be designed to describe the details of selection process.

### 2.5 Data extraction and management

Two reviewers will extract data from the selected studies independently. The following information will be extracted: first author's name, publication year, journal, study design, patient information, experimental intervention, control intervention, duration of follow-up, and outcomes. Any disagreement will be resolved by discussion with the third reviewer. If some important information is missing, we will contact original authors by email to request detailed information about the research.

### 2.6 Assessment of risk of bias

The Cochrane risk of bias assessment tool will be used to assess the risk of bias. Random sequence generation, allocation concealment, blinding of participants, and personnel, blinding of outcome assessment, incomplete outcome data, selective reporting, and other bias will be assessed by 2 reviews independently. A bias value of “high”, “unclear”, or “low” will be given for each domain. The rating results will be cross-checked and the difference will be solved by the third reviewer. Bias graph and graphic summary of risk of bias will be provided in the completed review.

### 2.7 Data synthesis and analysis

#### 2.7.1 Data synthesis

We will perform data synthesis using Review Manager 5.3 software (The Cochrane Collaboration, Software Update, Oxford, UK). Risk ratio with 95% confidence interval will be used for dichotomous variables. Mean difference with 95% confidence interval will be used for continuous variables. Heterogeneity will be examined using the I^2^ test. The I^2^ value > 50% means significant heterogeneity, and the random effects model will be used. Otherwise, the I^2^ value ≤ 50% means minor heterogeneity, and the fixed effects model will be utilized.

#### 2.7.2 Subgroup analysis

Subgroup analysis will be performed to explore the potential heterogeneity and inconsistency based on differences in participant characteristics, administration routes, dosage, and duration of intervention.

#### 2.7.3 Sensitivity analysis

To check the robustness and reliability of data analysis, sensitivity analysis will be applied. We will perform meta-analysis repeatedly after eliminating studies in low quality.

#### 2.7.4 Reporting bias

If ≥10 trials are included, publication bias will be assessed with funnel plot and Egger regression analysis.^[25,26]^

### 2.8 Ethics and dissemination

Ethical approval is not necessary because this study is based on literature analysis. The results of this study will be published in a peer-reviewed journal.

## 3 Discussion

This is the first systematic review and meta-analysis to conduct a comprehensive literature search and provide a systematic synthesis of current published data to explore the effectiveness of iron supplementation for PPD. Seven electronic literature databases will be searched to avoid missing any potential eligible studies. We will apply rigorous methodology to examine studies reporting iron supplementation for PPD. We believe that this systematic review and meta-analysis will provide clinical evidence for the effectiveness of iron supplementation for PPD, inform our understanding of the value of iron supplementation in improving PPD symptoms, and help clinicians to make better decisions regarding the appropriate role of iron supplementation as a part of prevention and treatment routines.

## Author contributions

**Conceptualization:** Yanran Tian, Zeyu Zheng, Chen Ma.

**Data curation:** Yanran Tian, Chen Ma.

**Formal analysis:** Zeyu Zheng, Chen Ma.

**Funding acquisition:** Zeyu Zheng.

**Investigation:** Yanran Tian, Chen Ma.

**Methodology:** Chen Ma.

**Project administration:** Zeyu Zheng.

**Resources:** Yanran Tian, Chen Ma.

**Software:** Yanran Tian, Chen Ma.

**Supervision:** Zeyu Zheng.

**Validation:** Zeyu Zheng.

**Visualization:** Yanran Tian, Chen Ma.

**Writing – review & editing:** Yanran Tian, Zeyu Zheng, Chen Ma.

**Writing – review & editing:** Yanran Tian, Zeyu Zheng, Chen Ma.
